# A plasmid-mediated type III secretion system associated with invasiveness and diarrheagenicity of *Providencia rustigianii*

**DOI:** 10.1128/mbio.02297-24

**Published:** 2024-09-09

**Authors:** Jayedul Hassan, Atsushi Hinenoya, Noritoshi Hatanaka, Sharda Prasad Awasthi, Goutham Belagula Manjunath, Nahid Rahman, Jyoji Yamate, Shota Nakamura, Daisuke Motooka, Akira Nagita, Shah M. Faruque, Shinji Yamasaki

**Affiliations:** 1 Graduate School of Life and Environmental Sciences, Osaka Prefecture University, Osaka, Japan; 2 Graduate School of Veterinary Science, Osaka Metropolitan University, Osaka, Japan; 3 Asian Health Science Research Institute, Osaka Metropolitan University, Osaka, Japan; 4 Osaka International Research Institute for Infectious Diseases, Osaka Metropolitan University, Osaka, Japan; 5 Research Institute for Microbial Diseases, Osaka University, Osaka, Japan; 6 Department of Pediatrics, Mizushima Central Hospital, Okayama, Japan; 7 School of Environment and Life Sciences, Independent University Bangladesh (IUB), Dhaka, Bangladesh; University of Texas Health Science Center, Houston, Texas, USA

**Keywords:** *Providencia*, T3SS, plasmid

## Abstract

We have recently described a clinical isolate of *Providencia rustigianii* strain JH-1 carrying the genes for cytolethal distending toxin (CDT) in a conjugative plasmid. A *cdtB* mutant of strain JH-1, which lost CDT activity, was still found to retain invasiveness and diarrheagenicity. The strain was subjected to phenotypic and genetic analyses including whole genome sequencing (WGS) to explore the genetic determinants of the observed invasiveness and diarrheagenic properties. Analysis and annotation of WGS data revealed the presence of two distinct type III secretion systems (T3SS) in strain JH-1, one of which was located on the chromosome designated as cT3SS (3,992,833 bp) and the other on a mega-plasmid designated as pT3SS (168,819 bp). Comparative genomic analysis revealed that cT3SS is generally conserved in *Providencia* spp. but pT3SS was limited to a subset of *Providencia* spp., carrying *cdt* genes. Strain JH-1 was found to invade HeLa cells and induce fluid accumulation with characteristic pathological lesions in rabbit ileal loops. Remarkably, these phenomena were associated with the pT3SS but not cT3SS. The plasmid could be transferred by conjugation from strain JH-1 to other strains of *P. rustigianii*, *Providencia rettgeri*, and *Escherichia coli* with concomitant transfer of these virulence properties. This is the first report of a functional and mobile T3SS in *P. rustigianii* and its association with invasiveness and diarrheagenicity of this bacterium. These data suggest that *P. rustigianii* and other CDT-producing *Providencia* strains might carry T3SS and exert their diarrheagenic effect by exploiting the T3SS nano-machinery.

**IMPORTANCE:**

The precise mechanism of virulence of *Providencia rustigianii* is unclear, although some strains produce cytolethal distending toxin as a putative virulence factor. We have detected the presence of a type III secretion system (T3SS) for the first time on a plasmid in a *P. rustigianii* strain. Plasmid-mediated T3SS seems to be directly involved in virulence of *P. rustigianii* and may serve as a means of horizontal transfer of T3SS genes. Our results may have implication in understanding the mechanism of emergence of new pathogenic strains of *P. rustigianii*.

## INTRODUCTION

Some strains of the genus *Providencia* belonging to different species are potentially pathogenic to humans (1–5). Among them, *Providencia alcalifaciens*, *Providencia rettgeri*, and *Providencia stuartii* show potential virulence properties including motility, adherence, and invasion, and produce protein toxin such as cytolethal distending toxin (CDT), which is considered as a key determinant of their enteropathogenicity (1–10). Although the molecular mechanism of pathogenicity of *Providencia* is not fully understood, invasiveness is considered as a major step in the mechanism of *P. alcalifaciens*-induced diarrhea (2). *P. alcalifaciens* was found to invade intestinal mucosal epithelium and produce localized bacterial clusters and actin condensation, leading to diarrhea (2). Certain *Providencia* strains produce CDT, which is suggested to be associated with inflammation and diarrhea in animal models and presumably in human (7, 11–14). In our previous study, we demonstrated that a *Providencia rustigianii* strain JH-1 isolated from a child with diarrhea produced functional CDT (5). To verify whether CDT production was associated with diarrheagenicity of this strain, we initially analyzed this strain JH-1 and its *cdt* gene-deleted derivative and found that *cdt* deletion did not alter its invasiveness in HeLa cells (Fig. S1). This result indicated the presence of possible other virulence determinants in *P. rustigianii* strain JH-1 besides CDT. The type III secretion system (T3SS) is a sophisticated nano-machinery used by several Gram-negative bacteria including *Salmonella enterica*, *Shigella* spp., *Vibrio parahaemolyticus*, *Pseudomonas aeruginosa*, and *Escherichia coli* for attachment, invasion, induction of diarrhea, and dissemination into the host (15–18). But the presence of T3SS and their association with pathogenicity of *Providencia* spp. are not clear. However, functional complementation of *S*. Typhimurium Inv/Mxi-Spa family T3SS translocation operon (*sicAsipBCD*) with its ortholog from *P. alcalifaciens* (19) indicated the presence of T3SS-related genes in *Providencia* and a possible involvement of T3SS with the virulence of this bacterium. Hence, this study was focused on the possible presence and function of T3SS-related genes in *P. rustigianii* stain JH-1. We found for the first time that *P. rustigianii* strain JH-1 carries two distinct T3SS gene clusters, one being on the chromosome (cT3SS) and the other on a large plasmid (pT3SS), and that the pT3SS but not cT3SS is directly associated with invasiveness and diarrheagenicity of *P. rustigianii* strain JH-1.

## MATERIALS AND METHODS

### Bacterial strains and cultural conditions

*P. rustigianii* strain JH-1 and different strains of *P. alcalifaciens*, *P. rettgeri*, and *E. coli* were used in this study (Table 1). Unless otherwise mentioned, the bacterial strains were routinely cultured in Luria Bertani (LB) broth (Becton, Dickinson and Co., Franklin Lakes, NJ) by aerobic incubation at 37°C with shaking (180 rpm).

**TABLE 1:** Bacterial strains used in this study

Bacteria	Strains	Characteristics^*a*^	Reference
*P. rustigianii*	JH-1	Clinical, CL^r^	(5)
	∆*cdtB*	∆*cdtB*::*bla,* Amp^r^, CL^r^	(5)
	∆c*spaL*	∆c*spaL,* CL^r^	This study
	∆p*spaL*	∆p*spaL*, CL^r^	This study
	∆*invF*	*∆invF::bla,* Amp^r^, CL^r^	This study
	∆c*spaL_*c*spaL*	∆c*spaL*/pBR322::c*spaL*, Amp^r^, Tet^r^	This study
	∆p*spaL_*p*spaL*	∆p*spaL*/pBR322::p*spaL*, Amp^r^, Tet^r^	This study
	∆c*spaL_*pBR322	∆c*spaL*/pBR322, Amp^r^, Tet^r^	This study
	∆p*spaL_*pBR322	∆p*spaL*/pBR322, Amp^r^, Cm^r^, Tet^r^	This study
	GTC1504^T^	Human, CL^r^	GCMR
*P. rettgeri*	GTC1263^T^	Unknown, CL^r^, Tet^r^	GCMR
	GTC1263^T^-TC ∆*cdtB*	GTC1263^T^/pJH-1 ∆*cdtB,* Amp^r^, CL^r^, Tet^r^	(5)
	GTC1263^T^-TC ∆p*spaL*	GTC1263^T^/pJH-1 ∆p*spaL,* Cm^r^, CL^r^, Tet^r^	This study
*P. alcalifaciens*	AH-31	Clinical, CL^r^	(14)
	AS-1	Clinical, CL^r^	(7)
	F90-2004	Clinical, CL^r^	(2)
	P6400	Clinical, CL^r^	Hinenoya et al. (unpublished)
*E. coli*	KC95	Cheese, Tet^r^	(20)
	KC95-TC ∆*invF*	KC95/pJH-1 ∆*invF,* Amp^r^, Tet^r^	This study
	C600		Laboratory strain
	JM109		Laboratory strain

^*a*^ Antimicrobial resistance that was considered during counter selection in conjugation experiment or acquired after mutation and/or conjugation was included in this column. Amp, ampicillin; CL, colistin; Cm, chloramphenicol; Tet, tetracycline; ^r^, resistant; GCMR, Gifu University Center for Conservation of Microbial Genetic Resource.

### Whole genome sequencing and annotation

Whole genome sequencing (WGS) was performed using PacBio RSII (Pacific Bioscience, California Inc., USA) and MiSeq (Illumina Inc., San Diego, CA) platforms according to the manufacturer’s instruction. Briefly, bacterial cells were collected from an overnight culture of the relevant strain by centrifugation (1,000 × *g* for 10 min at 4°C) followed by the extraction of genomic DNA using a Power Soil DNA Extraction Kit (MO BIO Laboratories, Carlsbad, CA). PacBio sequencing was performed by shearing 10 µg of DNA with g-TUBE (Covaris, Woburn, MA), and library was prepared using a DNA Template Prep Kit 1.0 (Pacific Bioscience) following the manufacturer’s instructions. For MiSeq sequencing, 500 ng of DNA was sheared using the Covaris S220 (Covaris) and the library was prepared using a KAPA Library Preparation Kit (Kapa Biosystems, Wilmington, MA). Identification of ORFs and annotation were done by RAST [Rapid Annotation using Subsystems Technology (21)] followed by cross checking with ORFs prepared manually with CLC Genomics Workbench 9.0 (QIAGEN, GmbH, Venlo, the Netherlands). Annotations were checked for their accuracy by BLAST search (http://www.ncbi.nlm.nih.gov/BLAST). The annotated sequences were registered to the DNA Data Bank of Japan with accession numbers AP018946 (chromosome) and AP018947 (plasmid).

### Comparative analysis of T3SS gene clusters

The sequence of T3SS gene clusters was extracted from the whole genome sequence of various strains including *P. rustigianii* (Acc. No.LR134189), *P. alcalifaciens* (Acc. No. CP023536), *P. stuartii* (Acc. No. CP119546), *P. rettgeri* (Acc. No. CP059348), and *P. heimbachae* (Acc. No. CP028384), which were downloaded from the GenBank (https://www.ncbi.nlm.nih.gov/). Linear comparison of these annotated sequences with the T3SS clusters of strain JH-1 was performed on Easyfig (http://mjsull.github.io/Easyfig/) version 2.1 to explore their genetic relatedness.

### Construction of mutants and preparation of the transconjugants

T3SS-deficient mutants of strain JH-1 were prepared by homologous recombination or site-directed mutagenesis following the protocol described in reference (19) with minor modifications. The primers used for the preparation of mutants are listed in Table S1. Briefly, the chromosomal T3SS (cT3SS)-deficient mutant (Δc*spaL*) and the plasmid T3SS (pT3SS)-deficient mutant (Δp*spaL*) were prepared by deleting the *spaL* gene on chromosome (c*spaL*) and plasmid (p*spaL*), respectively.

Complementation of the p*spaL* gene was performed by cloning the gene along with its putative promoter sequence into a plasmid vector pBR322 (New England Biolabs, Ipswich, MA), and introducing the modified plasmid into the respective mutant. Briefly, for the complementation of the p*spaL* gene, the gene with its 22 bp upstream and the putative promoter region located upstream of the *invF* gene (locus 117) were amplified by PCR and joined in tandem (promoter gene). The sequence of primers used containing appropriate restriction sites is provided in Table S1. For complementation of c*spaL*, the target gene was amplified from strain JH-1 using the appropriate forward and reverse primers. The relevant amplicons as described above were ligated into pBR322 at appropriate restriction sites by standard methods. The recombinant plasmids were used to transform the respective mutants (Δc*spaL* or Δp*spaL*) using a protocol described previously (22).

*P. rettgeri* strain GTC1263 transconjugants (Δc*dtB* or Δp*spaL*) were generated by conjugation using strain JH-1 derivatives (Δ*cdtB* or Δp*spaL*) as donors. Resulting transconjugants were confirmed to be GTC1263 construct by urease production test (strain GTC1263 was urease positive but strain JH-1 derivatives were negative) and Pulsed-Field Gel Electrophoresis (PFGE) typing using their *Sma*I-digested genomic DNA. Similarly, *E. coli* strain KC95 Δ*invF* was created by conjugation using pJH-1 ∆*invF* mutants (in which the *invF* gene on pJH-1 was replaced with the *bla* gene), as a donor, and was differentiated from the parental strains by a PCR assay targeting the *cdtB* gene and screening on MacConkey agar plates (donor strain produced colorless colonies whereas strain KC95 produced pinkish colonies).

### Cell invasion assay

Cell invasion was tested using HeLa cells by a gentamicin protection assay following the protocol described in reference (23) with minor modifications. HeLa cells were seeded in 24-well cell culture plates (5 × 10^4^ cells/well). The cells were maintained in minimum essential media (MEM) (Shimazu Diagnostics Corp., Tokyo, Japan) with 5% fetal bovine serum (FBS) (Thermo Fisher Scientific, Waltham, MA), 1% GlutaMax (Thermo Fisher Scientific), and 1% antibiotic cocktails (Thermo Fisher Scientific). After 24 h of pre-incubation, the HeLa cell monolayer was washed with Dulbecco's phosphate buffered saline (PBS, pH 7.4) and 1 mL of bacterial suspension of *P. rustigianii* JH-1 strains, including wild-type (WT), mutant, and their complementary strains, was added in each well to have a multiplicity of infection as 1.0. The bacterial suspensions were prepared from the log phase culture in LB broth. Bacterial cells were collected by centrifugation at 1,000 × *g* for 10 min at 4°C, washed once with PBS, and suspended in MEM (without antibiotic cocktails) at 5 × 10^4^ cells/ mL. After 3 h of infection, the cell monolayer was washed three times with PBS and incubated with MEM containing gentamicin (250 µg/mL) for 1 h. The cell monolayer was then washed three times with PBS and lysed with 0.1% Triton X-100 at room temperature (RT) for 10 min and diluted in PBS. Each dilution was plated onto LB agar plates for the enumeration of internalized bacteria. *Shigella flexneri* strain NK1983 and *E. coli* strain JM109 were used as positive and negative controls, respectively.

### Rabbit ileal loop test

Enterotoxicity of the strain JH-1 was determined by rabbit ileal loop test according to reference (3) with minor modifications. Briefly, bacteria were grown in LB broth at 37°C until log phase [optical density (OD) 600 = ~0.5] and collected by centrifugation at 3,800 × *g* at 4°C for 10 min. Bacterial pellets were suspended in PBS, and OD_600_ was adjusted to 1.0 (~ 2 × 10^7^ CFU/mL). One milliliter of the bacterial suspension (OD_600_ = 1.0 or 0.1) was injected into the 8-cm ileal loop of 8-week-old rabbits. One milliliter of the culture supernatant prepared from *Vibrio cholerae* strain CRC41 (cultured in AKI media at 37°C for 4 h of static followed by 4 h of vigorous shaking) was used as a positive control, and the same volume of PBS was used as a negative control. In enterotoxicity study with the T3SS-*spaL* mutants (JH-1 Δc*spaL*/Δp*spaL*), wild-type strain JH-1 was used as a positive control. The fluid accumulation (FA) ratio was calculated by dividing the volume of accumulated fluid (mL) by the length of the respective loop (cm). The experiment was performed at least three times to check reproducibility. All animal experiments were conducted with the approval of Animal Experiment Committee at Osaka Prefecture University (#30-151).

### Histopathological examination of the intestinal tissues

Tissue sections from the ileal loops challenged by bacteria were collected for histopathological examination. Dissected ileal loops were gently washed with PBS and fixed in 10% neutral buffered formalin. Tissue sections were further processed by a tissue processor for around 24 h. After fixation, tissues were embedded in paraffin wax, sectioned at 4 µM in thickness, and stained with hematoxylin and eosin for microscopic evaluation. Then, slides were observed, and images were prepared by microscopy using an Olyvia 2.9 slide viewer (Life Sciences Solutions, Olympus, Shinjuku, Tokyo, Japan).

### RNA isolation

Total RNA was extracted from *P. rustigianii* JH-1 strains (WT, mutants, and complemented strains) using TRIzol reagent (Thermo Fisher Scientific), according to the manufacturer’s instructions. Briefly, bacteria were cultured in LB broth at 37°C for around 6.5 h with shaking at 180 rpm and collected from 1 mL of the late log phase culture by centrifugation at 15,400 × *g* at 4°C for 5 min. Bacterial pellet was suspended in 1.0 mL TRIzol by pipetting and kept at RT for 5 min. Two hundred microliters of chloroform was added into the cell suspension and mixed with vigorous shaking for 15 s. The mixture was kept at RT for 7 min, and the upper aqueous phase was collected by centrifugation as described above. RNA was precipitated using isopropanol and washed with 75% ethanol. Isolated RNA pellet was dissolved in diethyl pyrocarbonate-treated water and kept at −80°C until use.

### DNase treatment and cDNA preparation

Five micrograms of RNA were treated with 2 U DNase I (1 U/µL, amplification grade, Thermo Fisher Scientific) at RT for 14 min. DNase activity was inactivated by adding 2 µL of 25 mM ethylenediaminetetraacetic acid (EDTA) (final conc. 2.5 mM), and residual DNase was further deactivated by heating at 65°C for 10 min. cDNA was prepared from 2 µg of the RNA using a High-Capacity RNA-to-cDNA Kit (Thermo Fisher Scientific) with an incubation at 37°C for 60 min followed by 95°C for 5 min. Synthesized cDNA were kept at −80°C until use.

### Quantitative real-time PCR (qRT-PCR)

Quantitative real-time PCR (qRT-PCR) was performed with about 10 ng of the synthesized cDNA following the SYBR Green method using a GoTaq qPCR master mix (2× master mix, Promega, Madison, WI). To determine the possible mechanism of *spaL*-mediated invasiveness, related expression of 11 genes encoding for homologs of T3SS needle inner membrane ring protein (SpaS), needle protein (PrgI), target-protein (SipC), chaperone (SpaK, SicA), effector protein (SipB, SipD), regulator (InvE), protein InvA, and ATPase (SpaL) was examined. Expression of *recA* was used as the internal control. The target genes and primers used in this study are summarized in Table S2. PCR conditions were 95°C for 30 s followed by 35 cycles of 95°C for 15 s and 60°C for 1 min in an ABI Prism 7500 sequence detection system (Thermo Fisher Scientific).

Relative gene expression was calculated from the ct values by the ΔΔCq method following the procedure described in reference (24). Briefly, expression of the gene was normalized with a non-target reference such as *recA* expression of the same strain to determine ΔCq (see the equations below). The ΔCq for each replicate was transformed to the ΔCq expression. Subsequently, the mean of the ΔCq of gene was normalized with that of control such as *P. rustigianii* strain JH-1 (WT) to find ΔΔCq expression.


$$\Delta Cq=Cq\;\left(Target)-(Reference\right)\;\Delta Cq\;expression=2^{-\Delta \Delta Cq}\;\Delta \Delta Cq=Normalize\;\Delta Cq\;to\;the\;control$$

Cq: CT value obtained from qPCR.

### Assay for horizontal transfer of pT3SS

Conjugation experiments were performed to examine the transferability of the pT3SS carrying plasmid (pJH-1) from *P. rustigianii* strain JH-1 to other enteric bacteria listed in Table S3, according to the procedure described in reference (25) with minor modifications. Briefly, a single colony of each donor and recipient was cultured in LB broth at 37°C for about 16 h with shaking at 180 rpm. Aliquot of the culture was inoculated in fresh LB broth (30:1) and incubated at 37°C until OD_600_ reaches 0.5–0.9. Hundred microliters of the culture of donor and recipient were mixed and centrifuged at 3,800 × *g* at 4°C for 2 min. The bacterial pellet was resuspended in 100 µL fresh LB broth and dropped onto an LB agar plate. After around 18 h culture incubation at 37°C, bacterial cells were collected by scraping and suspended in 5 mL PBS. Hundred-microliter aliquots of the 10-fold serially diluted suspension were spread onto LB agar plates containing counter selection antibiotics (Table 1). TCs were confirmed as the derivative of recipient strains by differential culture, biochemical examination, PCR, and PFGE with *Sma*I restriction digestion (5). Transformation efficiency was calculated by dividing the number of colonies obtained in the double selection plates (transconjugants) by the number of recipient strain on single-selection media.

### S1 nuclease PFGE (S1-PFGE) and southern hybridization

Presence and location of T3SS’s genes identified in *P. rustigianii* strain JH-1 were examined in other *Providencia* spp. by S1-PFGE followed by southern hybridization as described previously (5). Briefly, bacterial cells from log phase cultures were collected by centrifugation (1,000 × *g*, 10 min at 4°C) and suspended in cell suspension buffer [10 mM Tris-HCl (pH 7.2), 20 mM NaCl, and 100 mM EDTA]. The bacterial suspension was embedded in 0.5% Seakem gold agarose (Lonza Rockland, ME) followed by *in situ* lysis in cell lysis buffer [10 mM Tris-HCl (pH 7.2), 50 mM NaCl, 100 mM EDTA, 0.2% SDS, and 0.5% *N*-lauroylsarcosine] at 70°C for 90 min with gentle shaking followed by soaking in wash solution [20 mM Tris-HCl (pH 8.0), 50 mM EDTA] at 70°C for 15 min. Then, gel plugs were treated with proteinase K solution [0.8 mg/mL proteinase K (P8044-5G, Merck KGaA, Darmstadt, Germany), 100 mM EDTA, 0.2% SDS, and 1% *N*-lauroylsarcosine (Merck KGaA)] at 42°C for 18 h with gentle shaking followed by soaking in wash solution containing 1 mM PMSF (phenyl methyl sulfonyl fluoride, Merck KGaA) at 42°C for 18 h with gentle shaking followed by soaking in wash solution containing 1 mM PMSF (phenyl methyl sulfonyl fluoride, Merck KGaA) at 42°C for 1 h with gentle shaking. Finally, the gel plugs underwent three subsequent washing with fresh 10-times-diluted washing solution and stored at 4°C until use. Half of the gel plug containing genomic DNA was treated with 4 U of S1 nuclease (Thermo Fisher Scientific) at 37°C for 45 min in 200 µL of 30 mM sodium acetate buffer (pH 4.6) including 50 mM NaCl, 1.0 mM zinc acetate, and 5% (vol/vol) glycerol. The digested DNA were resolved on 1.0% pulsed-field certified agarose (Bio-Rad Laboratories, Hercules, CA) in 0.5× TBE [45 mM Tris, 45 mM boric acid, and 1 mM EDTA (pH 8.0)] buffer at 6 V/cm^2^, with a switch time of 2.2–54.2 s for 21 h using a CHEF Mapper (Bio-Rad). *Salmonella* Braenderup strain H9812 was used as a molecular size marker upon digestion with *Xba*I at 37°C for 2 h. The resolved DNA were transferred onto nylon membranes (PerkinElmer, Waltham, MA) followed by hybridization with ^32^P {[α-^32^P]-dCTP (111 TBq/ mmol) (Perkin Elmer)}-labeled DNA probes prepared from the c*spaL* or p*spaL* gene of strain JH-1 by a random priming method using a MultiPrime DNA Labeling System (Cytiva, Marlborough, MA). Radioactivity was visualized by the BAS FLA-3000 system (Cytiva).

## RESULTS

### T3SS and their organization on the chromosome and plasmid

**Fig 1:**
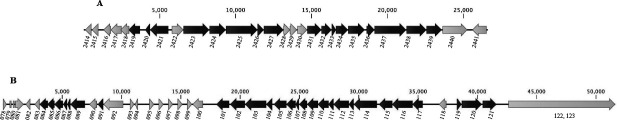
T3SS gene cluster in *P. rustigianii* strain JH-1. (**A**) T3SS gene cluster on chromosome (cT3SS); (**B**) T3SS gene cluster on the plasmid (pT3SS). ORFs are indicated by different shaded arrows. Dark arrows indicate putative T3SS-related genes while the light arrows for hypothetical or other genes. Number under each ORF indicated corresponding locus_tags (accession nos. AP018946–AP018947).

WGS revealed the sequence of a 3,992,833-bp chromosome and a plasmid of 168,819 bp with GC contents of 41.2% and 38.7%, respectively. Annotation of the chromosome and plasmid sequences showed 3,595 and 126 CDS, respectively. Two distinct T3SS-related gene clusters were found, one of which was on the chromosome (cT3SS) and the other on the plasmid (pT3SS), respectively. The cT3SS comprises a ~25-kb gene cluster and is similar to those of *Providencia* spp. registered in GenBank (accession nos. LR134189, CP023536, CP119546, CP059348, and CP028384; Fig. 1A). Therefore, the genes on the cT3SS cluster were named based on their homology to *Providencia* spp. (accession no. AP018946). On the other hand, the pT3SS comprises a ~35-kb gene cluster (Fig. 1B) and showed structural and deduced amino acid sequence homology (30%–70%) to those of T3SS1 of *Salmonella enterica* (accession no. CP0424431), and thus, the genes were named (accession no. AP018947).

### Comparative analysis of T3SS gene clusters

**Fig 2:**
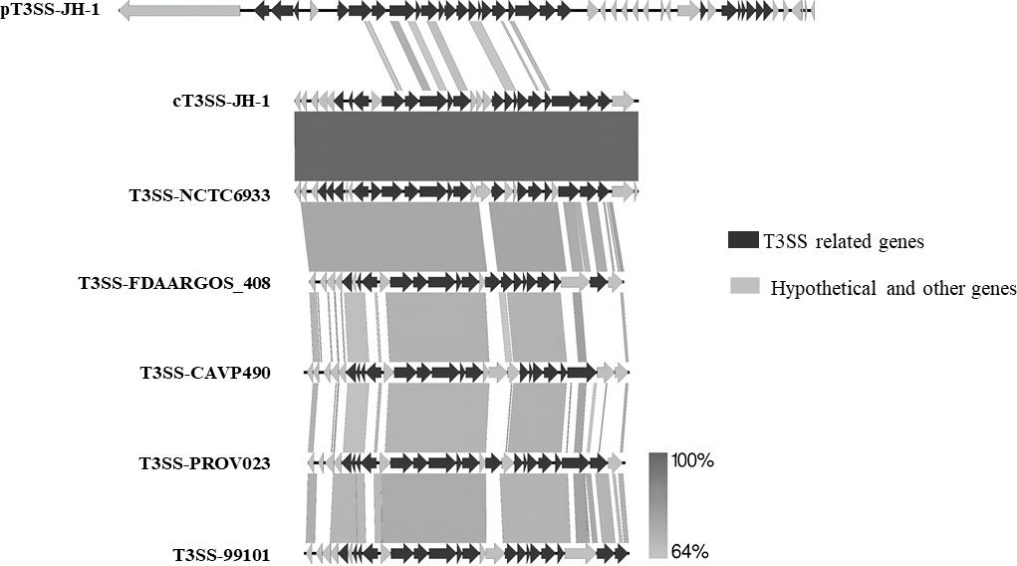
Comparative illustrations of T3SS in *P. rustigianii* strain JH-1 and other *Providencia* spp. pT3SS-JH-1 and cT3SS-JH-1, T3SS on the plasmid and chromosome of strain JH-1, respectively; T3SS-NCTC6933, T3SS in *P. rustigianii* strain NCTC6933 (accession no. LR134189); T3SS-FDAARGOS_408, *P. alcalifaciens* strain FDAARGOS_408 (accession no. CP023536); T3SS-CAVP490, *P. stuartii* strain CAVP490 (accession no. CP119546); T3SS-PROV023, *P. rettgeri* strain PROV023 (accession no. CP059348); T3SS-99101, *P. heimbachae* strain 99101 (accession no. CP028384). Figure was prepared by using Easyfig (http://mjsull.github.io/Easyfig/). The color gradient bar indicates blast identity percentage between the loci.

Comparative analysis of the T3SS gene clusters (performed using Easyfig software) revealed that the chromosomal T3SS (cT3SS) of strain JH-1 is almost identical to that of *P. rustigianii* strain NCTC6933 (accession no. LR134189) and showed ≥64% similarities to that of *P. alcalifaciens* strain FDAARGOs_408 (accession no. CP023536), *P. stuartii* strain CAVP490 (accession no. CP119546), *P. rettgeri* strain PROV023 (accession no. CP059348), and *P. heimbachae* strain 99101 (accession no. CP028384) (Fig. 2). On the other hand, the sequence of the pT3SS gene cluster was unique with very little homology with the other T3SS gene clusters included in this analysis (Fig. 2). However, pT3SS genes of strain JH-1 showed similar genetic arrangement with various levels of nucleotide and amino acid homology with the T3SS-1 of *S*. Typhimurium (Fig. S2). Further analysis revealed that cT3SS belongs to Esc family T3SS and pT3SS belongs to the Inv/Mxi-Spa family.

### Association of T3SSs with the invasiveness of strain JH-1

**Fig 3:**
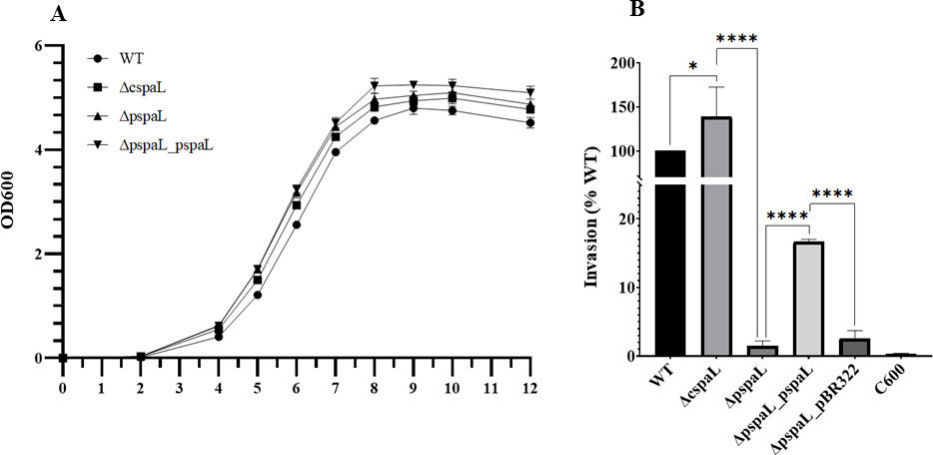
Invasion efficiency of *P. rustigianii* strain JH-1 (WT, ∆p*spaL*, ∆c*spaL*, and p*spaL* complemented strains) to HeLa cells. (**A**) Growth pattern of the strain JH-1 wild-type and *spaL* mutants and complemented strains (∆c*spaL*, ∆p*spaL*, and ∆p*spaL_*p*spaL*) at 37°C with shaking at 180 rpm. (**B**) Invasion of HeLa cells by *P. rustigianii* strain JH-1. WT, wild type; ∆c*spaL* and ∆p*spaL*, *spaL* gene mutants of cT3SS (chromosome) and pT3SS (plasmid) of strain JH-1, respectively; C600, *E. coli* strain C600 (negative control). * and ****, values are significantly different between the strains at *P* ≥ 0.01 and *P* ≥ 0.0001, respectively. One-way analysis of variance (ANOVA) was performed to determine the difference between the means of WT, ∆c*spaL*, and ∆p*spaL,* whereas unpaired *t*-test was performed for ∆p*spaL*, ∆p*spaL*_p*spaL*, and ∆p*spaL*_pBR322. Statistical analysis and image preparation were performed on GraphPad Prism 10.2.3 (403).

To elucidate the association of T3SSs with invasiveness, *spaL* gene mutants (Δc*spaL* and Δp*spaL*) of strain JH-1 were used. In addition, to rule out the possible association of CDT, invasiveness of strain JH-1 Δ*cdtB* was also determined. Although no significant difference was observed in invasiveness between the WT and Δ*cdtB* mutant (Fig. S1), invasiveness of the *spaL* gene mutants was found to be significantly different from that of WT strain in HeLa cells (Fig. 3B). Mutation in the *spaL* carried on the plasmid (Δp*spaL*) was found to decrease invasiveness, but in chromosomal *spaL* mutant (Δc*spaL*), the invasiveness in HeLa cells increased significantly compared with that of the WT strain (Fig. 3B). Invasiveness of Δp*spaL* could be partially restored in the p*spaL*-completed strain Δp*spaL*_p*spaL* (Fig. 3B). These results suggest that not CDT but T3SSs were associated with the invasiveness of the strain JH-1.

### Expression of pT3SS genes in *spaL* mutants of strain JH-1

**Fig 4:**
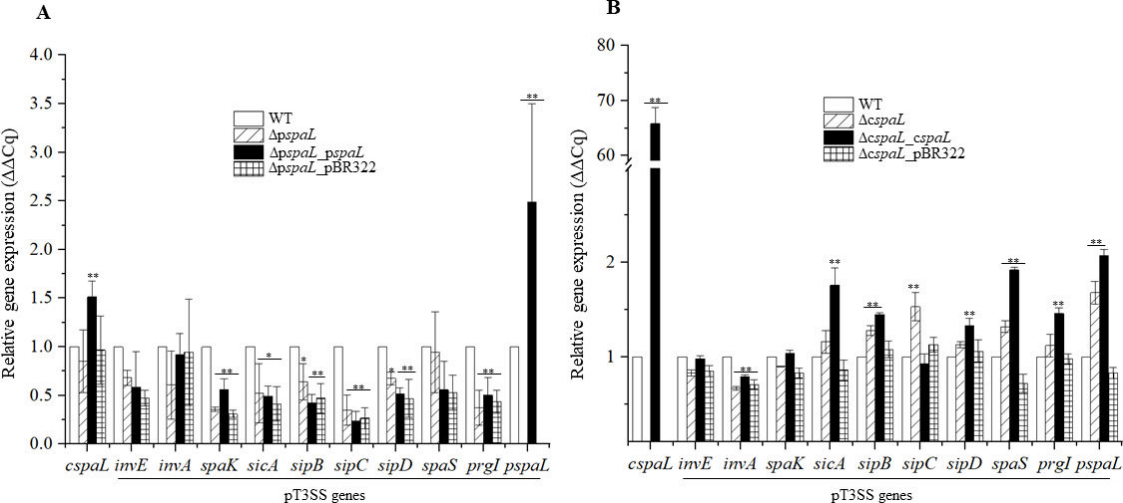
Relative expression of pT3SS genes in strain JH-1 (∆p*spaL* and ∆c*spaL*). (A and B) Relative expression of pT3SS genes in ∆p*spaL* and ∆c*spaL*. Gene expression was determined using qRT-PCR, and relative expression was calculated by ∆∆Cq method. *recA* was used as the reference gene, and wild-type *P. rustigianii* strain JH-1 was used as the control strain for the calculation of ∆∆Cq. * and **, values are significantly different from the WT at *P* ≤ 0.05 and *P* ≤ 0.01, respectively. Statistical analysis was performed by comparing means with one-way ANOVA on GraphPad Prism 10.2.3 (403).

Relative expressions of pT3SS genes were determined to understand possible molecular mechanisms of decrease or increase in invasiveness of *spaL* gene mutants. Expression of p*spaL* and c*spaL* was diminished in the respective mutants. All the pT3SS genes examined were downregulated in Δp*spaL*, with significant downregulation of p*spaK*, p*sicA*, p*sipBCD*, and p*prgI* genes, while c*spaL* gene expression remained unaffected (Fig. 4A). Conversely, the expression of p*spaL* and some of downstream (p*sipBC* and p*spaS*) genes was significantly upregulated in Δc*spaL* (Fig. 4B). Although there was a significant downregulation in the relative expression of p*invA*, no significant changes were observed in the expression of p*invE* and p*spaK* genes in Δc*spaL* (Fig. 4B). Complementation of p*spaL* in Δp*spaL* restored the expression of complemented fragment and partially restored the expression of p*invA* and p*spaK* genes. However, it failed to complement the expression of other genes affected by its deletion, such as p*sicA*, p*sipBCD*, and p*prgI* genes (Fig. 4A), which might be associated with the partial complementation of invasiveness in the Δp*spaL*_*pspaL* strain. On the other hand, complementation of the c*spaL* gene restored c*spaL* expression and influenced the expression of other tested genes in Δc*spaL* (Fig. 4B).

### Enterotoxicity of strain JH-1

**Fig 5:**
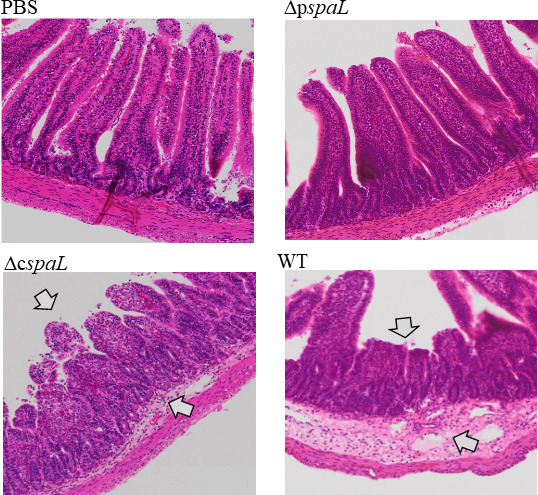
Histopathological changes in the rabbit ileal loops after 16 h of challenge with 2 × 10^7^ CFU of *Providencia rustigianii* strain JH-1 (WT, ∆c*spaL*, and ∆p*spaL*). PBS and ∆p*spaL* did not cause any histopathological changes, but the WT and ∆c*spaL* induced characteristic changes (arrows) including thickening of the lamina propria with marked neutrophil infiltration, flattened villi, and dilation of lymph vessels. Pictures were prepared by virtual microscopy using OlyVIA 2.9 slide viewer (5× magnification).

Enterotoxicity of strain JH-1 was analyzed by rabbit ileal loop assay. Strain JH-1 caused fluid accumulation in the rabbit ileal loops indicating its potential to cause diarrhea. On the other hand, whereas the Δp*spaL* strain lost the ability to cause fluid accumulation, the Δc*spaL* strain retained the ability to cause fluid accumulation, suggesting that the pT3SS but not cT3SS was directly associated with the fluid accumulation in the ileal loops of rabbits (Table 2; Fig. S3). Furthermore, light microscopic examination revealed characteristic histopathological changes in the ileal sections challenged with WT and Δc*spaL* strains but not with Δp*spaL* strain and PBS, used as a control. Histopathological changes included flattened villi, thickened lamina propria accompanied with neutrophil infiltration, and dilatation of the lymph vessels (Fig. 5).

**TABLE 2:** Fluid accumulation in the rabbit ileal loops induced by *Providencia rustigianii* strain JH-1

Strains (genotypes)	Challenge dose (CFU/mL)	^*c*^FA ratio (Avr. ± SD)
JH-1 (WT^*a*^)	~ 2.0 × 10^7^	0.7 ± 0.3
JH-1 (∆c*spaL*)	~ 2.0 × 10^7^	0.5 ± 0.2
JH-1 (∆p*spaL*)	~ 2.0 × 10^7^	0.0 ± 0.0
*V. cholerae* O1 CRC-41^*b*^	1 mL culture supernatant	2.0 ± 0.4
PBS	1 mL	0.0 ± 0.0

^*a*^ WT, wild type.

^*b*^ Bacteria were cultured in AKI media (4 h static + 4 h shaking) at 37°C followed by collection of supernatant and filtered through a 0.22-µm-pore-size syringe filter.

^*c*^ FA, fluid accumulation was calculated by dividing the accumulated fluid (mL) with the length (cm) of the respective loop.

### Distribution of T3SS genes similar to strain JH-1 in another *Providencia* spp.

The possible presence of T3SS genes similar to that of strain JH-1 was explored in other *Providencia* spp. by S1-PFGE followed by southern hybridization using ^32^P-labeled probe prepared from the *spaL* gene of cT3SS (c*spaL*)/pT3SS (p*spaL*) of strain JH-1. Sequences homologous to c*spaL* were detected on the chromosome of *cdt* gene-positive/negative *P. rustigianii* strains and in *P. alcalifaciens* strains AH-31 and AS-1 (Fig. S4). On the other hand, only *cdt* gene-positive *P. rustigianii* strain JH-1 and *P. alcalifaciens* strains AH-31, AS-1, F90-2004, and P6400 were positive for a plasmid-borne p*spaL* gene in the hybridization assays (Fig. S4). Whole genome sequencing and analysis of strain AH-31 revealed the presence of the pT3SS gene cluster similar to that of strain pJH-1 (Fig. S5), indicating that the strains, which were positive in hybridization assays, possibly also carry similar pT3SS-related genes on their plasmids.

### Expression of invasiveness by the transconjugants

**Fig 6:**
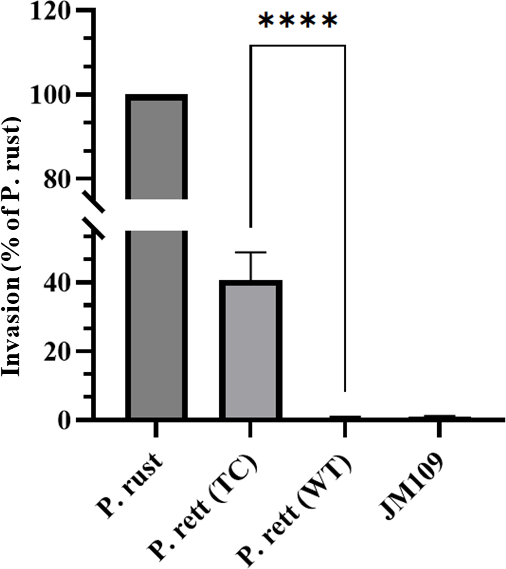
Invasiveness of *P. rettgeri* strain GTC1263 upon acquisition of pJH-1 through conjugation. P. rett (WT), *P. rettgeri* strain GTC1263; P. rett (TC), *P. rettgeri* strain GTC1263 with pJH-1 (∆*cdtB*); P. rust, *P. rustigianii* strain JH-1 (positive control); JM109, *E. coli* strain JM109 (negative control). ****, values are significantly different between P. rett (TC) and P. rett (WT) (*P* ≤ 0.0001). Statistical analysis with one-way ANOVA and image preparation was performed on GraphPad Prism 10.2.3 (403).

Plasmid pJH-1 carrying pT3SS gene clusters could be transferred to *P. rustigianii* strain GTC1504, *P. rettgeri* strain GTC1263, and *E. coli* strains KC95 with transformation efficiency of ≤10^−5^ for *P. rustigianii* and *P. rettgeri* and ≤10^−3^ for *E. coli*, respectively (Table S3; Figs. S6 and S7). Transconjugants obtained from *P. rettgeri* strain GTC1263 (GTC1263^T^-TC Δ*cdtB*) were further examined for invasiveness and found to invade HeLa cells while the wild-type strain GTC1263 was non-invasive (Fig. 6).

## DISCUSSION

T3SS is a complex nano-machinery comprised of more than 20 different proteins belonging to three categories including structural proteins, effector proteins, and chaperones (26). Several species of Gram-negative bacteria such as *Yersinia* spp., *Salmonella enterica* serovar Typhimurium, *Shigella flexneri*, *Vibrio* spp., and enteropathogenic *E. coli* are known to use T3SS to introduce effector proteins or virulence factors directly from the bacteria into the host cell cytoplasm leading to changes in the host cell to facilitate ultrastructural alterations, bacterial invasion, persistence, and dissemination in the host cell (15, 17). Thus, acquisition of T3SS provides increased fitness to the bacteria, and mobility of T3SS through horizontal transfer is highly significant in the evolution of pathogenic bacteria. As discussed below, in the present study, we have identified a conjugative plasmid, which carries a functional T3SS. In this study, WGS analysis revealed the presence of two non-homologous T3SS gene clusters in *P. rustigianii* strain JH-1, one on the chromosome (cT3SS) and another on a plasmid (pT3SS), which is the first report of plasmid-borne T3SS in genus *Providencia*. The genetic organization of cT3SS was similar to that of another *Providencia* strain reported, but pT3SS is not similar to any of the T3SS reported earlier in the genus *Providencia*. Interestingly, in this study, we detected pT3SS-like sequences in a particular sub-set of *Providencia* carrying *cdt* genes. Furthermore, we demonstrated horizontal transfer of pT3SS-carrying plasmid to other related enteric pathogens. These findings suggest that cT3SS is conserved, but pT3SS has been acquired by certain *Providencia* strains through plasmid-mediated horizontal transfer events.

Several bacterial pathogens including *Salmonella* spp. and *Vibrio* spp. carry two different T3SS gene clusters having distinct functions (27–30). *S*. Typhimurium carries two T3SSs; T3SS1 on the pathogenicity island SPI-1 belongs to the Inv/Mxi-Spa family and is associated with the invasion and biogenesis of *Salmonella* containing vacuole (SCV), while T3SS2 on the SPI-2 belongs to the Esc family and is responsible for the maintenance or sustaining of the SCV (29, 30). Similarly, in this study, we found that *P. rustigianii* strain JH-1 carries two T3SS gene clusters; one on the chromosome (cT3SS) belongs to the Esc family, while the other (pT3SS) on the plasmid belongs to the Inv/Mxi-Spa family. Although the cT3SS did not show any homology with that of *S*. Typhimurium, the pT3SS has similar genetic arrangement and 30%–80% amino acid homology with that of T3SS1 in *S*. Typhimurium. In addition, the pT3SS was found to be associated with the invasiveness and diarrheagenicity of strain JH-1 similar to that of T3SS1 in *S*. Typhimurium. However, their distinct nucleotide sequences suggest that T3SS in *Providencia* is novel, although it has structural and functional similarities to that of T3SS1 in *S*. Typhimurium. This can also explain the functional complementation of *Salmonella* invasion chaperon (SicA) with ortholog from *P. alcalifaciens* (19). Although, the influence of the cT3SS on the virulence of strain JH-1 is not precisely understood, increased invasiveness was observed in the c*spaL* mutant. Gene expression through qRT-PCR revealed an increased expression of pT3SS genes in the ∆c*spaL* strain indicating a possible suppression effect of cT3SS over pT3SS on the wild-type strain. However, we do not have any direct evidence on the interaction between cT3SS and pT3SS, which leads to the suppressive effects and hence is worthy of further investigation.

This study also demonstrated that pT3SS is functional in *Providencia rustigianii* strain JH-1. Interestingly, pT3SS was found to be present only in the clinical strains (JH-1, AS-1, AH-31, F-90-2004, and P-6400) but not in 119 non-clinical strains suggesting possible relevance of pT3SS to pathogenicity in humans. In addition, mobility of the pT3SS carrying plasmid to related enteric pathogen and observed invasiveness by the transconjugants may represent a threat of emergence of highly virulent strains through acquisition of plasmid-borne T3SS. On the other hand, cT3SS has been detected both in clinical and in non-clinical isolates of *Providencia* spp. indicating that cT3SS is conserved in this genus and that cT3SS alone might not be sufficient to cause disease in humans. Notably, deletion of c*spaL* lead to the increased invasiveness of the strain JH-1, but the ability of fluid accumulation was slightly reduced, indicating a potential role of cT3SS on the intracellular survivability of the bacterium as reported for T3SS2 in *S*. Typhimurium (29, 30). It is not clear at this time why deletion of c*spaL* led to the observed increased invasiveness. However, it may have been caused directly or indirectly due to an increased transcription of p*sipB* and p*sipC*, encoding translocators (SipB and SipC). To clarify the role of cT3SS in pathogenicity, especially its interaction with the pT3SS and association with intracellular life of *Providencia* spp., further studies are necessary.

In this study, we have demonstrated the functionality of the T3SSs by deleting *spaL* genes, which encodes for ATPase, possibly involved in energizing secretion events and substrate recognition (31). However, it’s important to identify the effector proteins and their precise roles to further understand the role of T3SSs in *Providencia* spp. Besides, identification of the effector proteins responsible for those phenotypes may lead to the development of possible drugs targeting the T3SSs.

This study also has certain limitations, as we could not explain precisely why deletion of c*spaL* enhanced the invasiveness of strain JH-1. However, we observed that expression of the p*sipBC* gene was also enhanced, which may be associated with invasion enhancement. Nevertheless, further investigation will be necessary to more clearly understand the mechanism. In addition, complementation of invasiveness was partial in Δp*spaL*_p*spaL*, which might be related to the downregulation of p*sipBCD* translocons; however, further investigations are also warranted to uncover the exact mechanisms. Notably, the partial recovery of invasion by Δp*spaL*_p*spaL* (Fig. 3B) and invasion by the transconjugant (*P. rettgeri* with pJH-1) clearly indicate that pT3SS is indeed associated with invasiveness and thus is an important virulence factor in *P. rustigianii* strain JH-1. Despite these limitations, this study provides a comprehensive information on the molecular mechanism of invasion and diarrhea induction by *P. rustigianii*, paving the way for further studies on the molecular mechanism of gastroenteritis caused by another *Providencia* spp. beyond *P. rustigianii per se*.

## SUPPLEMENTAL MATERIAL

Supplemental material mbio.02297-24-s0001.pdf Supplemental tables and figures.

ASM does not own the copyrights to Supplemental Material that may be linked to, or accessed through, an article. The authors have granted ASM a non-exclusive, world-wide license to publish the Supplemental Material files. Please contact the corresponding author directly for reuse.

## References

[1] Haynes J, Hawkey PM. 1989. Providencia alcalifaciens and travellers’ diarrhoea. BMJ 299:94–95. doi:10.1136/bmj.299.6691.94-a PMC1837104 2504344

[2] Albert MJ, Alam K, Ansaruzzaman M, Islam MM, Rahman AS, Haider K, Bhuiyan NA, Nahar S, Ryan N, Montanaro J, Mathan MM. 1992. Pathogenesis of Providencia alcalifaciens-induced diarrhea. Infect Immun 60:5017–5024. doi:10.1128/iai.60.12.5017-5024.1992 1452332 PMC258271

[3] Murata T, Iida T, Shiomi Y, Tagomori K, Akeda Y, Yanagihara I, Mushiake S, Ishiguro F, Honda T. 2001. A large outbreak of foodborne infection attributed to Providencia alcalifaciens. J Infect Dis 184:1050–1055. doi:10.1086/323458 11574921

[4] Tumbarello M, Citton R, Spanu T, Sanguinetti M, Romano L, Fadda G, Cauda R. 2004. ESBL-producing multidrug-resistant Providencia stuartii infections in a university hospital. J Antimicrob Chemother 53:277–282. doi:10.1093/jac/dkh047 14688041

[5] Hassan J, Awasthi SP, Hatanaka N, Hoang PH, Nagita A, Hinenoya A, Faruque SM, Yamasaki S. 2023. Presence of functionally active cytolethal distending toxin genes on a conjugative plasmid in a clinical isolate of Providencia rustigianii. Infect Immun 91:e0012122. doi:10.1128/iai.00121-22 37158737 PMC10269090

[6] O’Hara CM, Steigerwalt AG, Green D, McDowell M, Hill BC, Brenner DJ, Miller JM. 1999. Isolation of Providencia heimbachae from human feces. J Clin Microbiol 37:3048–3050. doi:10.1128/JCM.37.9.3048-3050.1999 10449504 PMC85453

[7] Shima A, Hinenoya A, Asakura M, Sugimoto N, Tsukamoto T, Ito H, Nagita A, Faruque SM, Yamasaki S. 2012. Molecular characterizations of cytolethal distending toxin produced by Providencia alcalifaciens strains isolated from patients with diarrhea. Infect Immun 80:1323–1332. doi:10.1128/IAI.05831-11 22252871 PMC3318424

[8] Shima A, Hinenoya A, Samosornsuk W, Samosornsuk S, Mungkornkaew N, Yamasaki S. 2016. Prevalence of Providencia strains among patients with diarrhea and retail meats in Thailand. Jpn J Infect Dis 69:323–325. doi:10.7883/yoken.JJID.2015.224 26370430

[9] Kurmasheva N, Vorobiev V, Sharipova M, Efremova T, Mardanova A. 2018. The potential virulence factors of Providencia stuartii: motility, adherence and invasion. Biomed Res Int 2018:3589135. doi:10.1155/2018/3589135 29682537 PMC5841065

[10] Hassan J, Awasthi SP, Hatanaka N, Okuno K, Hoang PH, Nagita A, Hinenoya A, Yamasaki S. 2019. Development of a multiplex PCR targeting eae, stx and cdt genes in genus Escherichia and detection of a novel cdtB gene in Providencia rustigianii. Pathog Dis 76:ftz002. doi:10.1093/femspd/ftz002 30657893

[11] Okuda J, Fukumoto M, Takeda Y, Nishibuchi M. 1997. Examination of diarrheagenicity of cytolethal distending toxin: suckling mouse response to the products of the cdtABC genes of Shigella dysenteriae. Infect Immun 65:428–433. doi:10.1128/iai.65.2.428-433.1997 9009292 PMC174612

[12] Pandey M, Khan A, Das SC, Sarkar B, Kahali S, Chakraborty S, Chattopadhyay S, Yamasaki S, Takeda Y, Nair GB, Ramamurthy T. 2003. Association of cytolethal distending toxin locus cdtB with enteropathogenic Escherichia coli isolated from patients with acute diarrhea in Calcutta, India. J Clin Microbiol 41:5277–5281. doi:10.1128/JCM.41.11.5277-5281.2003 14605183 PMC262502

[13] Young VB, Knox KA, Pratt JS, Cortez JS, Mansfield LS, Rogers AB, Fox JG, Schauer DB. 2004. In vitro and in vivo characterization of Helicobacter hepaticus cytolethal distending toxin mutants. Infect Immun 72:2521–2527. doi:10.1128/IAI.72.5.2521-2527.2004 15102759 PMC387909

[14] Hinenoya A, Naigita A, Ninomiya K, Asakura M, Shima K, Seto K, Tsukamoto T, Ramamurthy T, Faruque SM, Yamasaki S. 2009. Prevalence and characteristics of cytolethal distending toxin-producing Escherichia coli from children with diarrhea in Japan. Microbiol Immunol 53:206–215. doi:10.1111/j.1348-0421.2009.00116.x 19714857

[15] Hueck CJ. 1998. Type III protein secretion systems in bacterial pathogens of animals and plants. Microbiol Mol Biol Rev 62:379–433. doi:10.1128/MMBR.62.2.379-433.1998 9618447 PMC98920

[16] Frankel G, Phillips AD, Trabulsi LR, Knutton S, Dougan G, Matthews S. 2001. Intimin and the host cell – is it bound to end in Tir(s)? Trends Microbiol 9:214–218. doi:10.1016/s0966-842x(01)02016-9 11336837

[17] Coburn B, Sekirov I, Finlay BB. 2007. Type III secretion system and disease. Clin Microbiol Rev 20:535–549. doi:10.1128/CMR.00013-07 17934073 PMC2176049

[18] Chowdhury G, Das B, Kumar S, Pant A, Mukherjee P, Ghosh D, Koley H, Miyoshi S-I, Okamoto K, Paul A, Dutta S, Ramamurthy T, Mukhopadyay AK. 2023. Genomic insights into extensively drug-resistant Pseudomonas aeruginosa isolated from a diarrhea case in Kolkata, India. Future Microbiol 18:173–186. doi:10.2217/fmb-2022-0140 36916516

[19] Klein JA, Dave BM, Raphenya AR, McArthur AG, Knodler LA. 2017. Functional relatedness in the Inv/Mxi-Spa type III secretion system family. Mol Microbiol 103:973–991. doi:10.1111/mmi.13602 27997726

[20] Ombarak RA, Hinenoya A, Elbagory A-R, Yamasaki S. 2018. Prevalence and molecular characterization of antimicrobial resistance in Escherichia coli Isolated from raw milk and raw milk cheese in Egypt. J Food Prot 81:226–232. doi:10.4315/0362-028X.JFP-17-277 29323530

[21] Aziz RK, Bartels D, Best AA, DeJongh M, Disz T, Edwards RA, Formsma K, Gerdes S, Glass EM, Kubal M, et al.. 2008. The RAST Server: rapid annotations using subsystems technology. BMC Genomics 9:75. doi:10.1186/1471-2164-9-75 18261238 PMC2265698

[22] Das S, Dash HR. 2015. Microbial biotechnology- A laboratory manual for bacterial systems. Springer India.

[23] Elsinghorst EA. 1994. Measurement of invasion by gentamicin resistance. Meth Enzymol 236:405–420. doi:10.1016/0076-6879(94)36030-8 7968625

[24] Haimes J, Kelley M. 2014. Demonstration of a ∆∆Cq calculation method to compute relative gene expression from qPCR data. Dharmacon, A Horizon discovery group company, Lafayette, CO, USA.

[25] Hardiman CA, Weingarten RA, Conlan S, Khil P, Dekker JP, Mathers AJ, Sheppard AE, Segre JA, Frank KM. 2016. Horizontal transfer of carbapenemase-encoding plasmids and comparison with hospital epidemiology data. Antimicrob Agents Chemother 60:4910–4919. doi:10.1128/AAC.00014-16 27270289 PMC4958172

[26] Deng W, Marshall NC, Rowland JL, McCoy JM, Worrall LJ, Santos AS, Strynadka NCJ, Finlay BB. 2017. Assembly, structure, function and regulation of type III secretion systems. Nat Rev Microbiol 15:323–337. doi:10.1038/nrmicro.2017.20 28392566

[27] Ibarra JA, Steele-Mortimer O. 2009. Salmonella--the ultimate insider. Salmonella virulence factors that modulate intracellular survival. Cell Microbiol 11:1579–1586. doi:10.1111/j.1462-5822.2009.01368.x 19775254 PMC2774479

[28] Park KS, Ono T, Rokuda M, Jang MH, Okada K, Iida T, Honda T. 2004. Functional characterization of two type III secretion systems of Vibrio parahaemolyticus. Infect Immun 72:6659–6665. doi:10.1128/IAI.72.11.6659-6665.2004 15501799 PMC523034

[29] Galán JE, Curtiss R. 1989. Cloning and molecular characterization of genes whose products allows Salmonella Typhimurium to penetrate tissue culture cells. Proc Natl Acad Sci U S A 86:6383–6387. doi:10.1073/pnas.86.16.6383 2548211 PMC297844

[30] Steele-Mortimer O, Brumell JH, Knodler LA, Méresse S, Lopez A, Finlay BB. 2002. The invasion-associated type III secretion system of Salmonella enterica serovar Typhimurium is necessary for intracellular proliferation and vacuole biogenesis in epithelial cells. Cell Microbiol 4:43–54. doi:10.1046/j.1462-5822.2002.00170.x 11856172

[31] Majewski DD, Worrall LJ, Hong C, Atkinson CE, Vuckovic M, Watanabe N, Yu Z, Strynadka NCJ. 2019. Cryo-EM structure of the homohexameric T3SS ATPase-central stalk complex reveals rotary ATPase-like asymmetry. Nat Commun 10:626. doi:10.1038/s41467-019-08477-7 30733444 PMC6367419

