# Supplementary material for: A plasmid-mediated type III secretion system associated with invasiveness and diarrheagenicity of Providencia rustigianii
Source: mBio. 2024 Sep 9;15(10):e02297-24. doi: 10.1128/mbio.02297-24 (PMC11481499; doi:10.1128/mbio.02297-24)
Supplement: Supplemental material — Supplemental tables and figures. [file mbio.02297-24-s0001.pdf]

Supplementary Table 1. Primers used for the preparation of mutants and complemented strains in this study

Primer's name	Sequence (5' – 3')	Purpose	Reference
cUpflank-cATPMu	CAA CAC GAG ATA TTA ACG TG	Preparation of Δ*cspaL*	This study
cJH-1-ATP-XhoI-F1	ATA TCT CGA GTG AAG TCT CTG AAA TGG TGA		
cJH-1-ATP-Up-R1	TCA TGA TGC TAG GTC AAT GAG TTA CAC TAA TTG TG		
cJH-1-ATP-down-F1	AAT TAG TGT AAC TCA TTG ACC TAG CAT CAT GAT GA		
cJH-1-ATP-NotI-R1	ATA TGC GGC CGC GGA CTA ACA GTT CCT CAG		
cJH-1-ATPsyn-F	ACA TCC AGT GCG CAT TCA		
cJH-1-ATPsyn-R	ATG TTC TGG TGT AGT CAC		
pUpflank-pATPMu	CTG AAC ACC ACG TTG ATG	Preparation of Δ*pspaL*	
pJH-1-ATP (CamR)-XhoI-F1	ATA TCT CGA GAC GTT CCT CTG AGT TAG AG		
pspaL-OLP-R1	GTCATTATTACGATGAGAAAATTAGGAGTTTGATTAATC		
pspaL-OLP-F1	TCAAACTCCTAATTTTCTCATCGTAATAATGACTGACA		
pJH-1-ATP (CamR)-NotI-R1	ATA TGC GGC CGC GAG TTA CCG ATA GCG CAT		
pATPsyn-F	CCG TAA GGT ATC TGA GAA		
pATPsyn-R	CTC AGC GAA TAT CTG GAC		
cspaL-Compl-F	ATA TCT GCA GAT CGA GTA TCC ACC TCG T	Preparation of Δ*cspaL*_*cspaL*	
cspaL-Compl-R	ATA TAG TAC TCT GTA CTC TTT CGA GTC G		
pspaL-compEcoRI-F1	ATA TGA ATT CAA TCG GGA GTT AGC CTG ATG	Preparation of Δ*pspaL*_*pspaL*	
pspaL-compOLP-R1	TAA TGA CTG ACA AAA ATT TCA AAT CAT AAT CAA TTT TAA C		
pspaL-compOLP-F1	ATT GAT TAT GAT TTG AAA TTT TTG TCA GTC ATT ATT AC		
pspaL-compHindIII-R1	ATA TAA GCT TTT ACG AAA GGG CAT ACA TTC		
XhoI(InvF)-F1	ATA TCT CGA GTC ACT CAT TGA AGC TTG T	Preparation of Δ*invF*-homolog	
Up(InvF)-R1	CAT GAG CGG ATA CAT ATT TGA ATG AAC GAA CAC CAC CAT AAT		
Up(InvF-bla)-F1	ATT ATG GTG GTG TTC GTT CAT TCA AAT ATG TAT CCG CTC ATG		
Down(InvF-bla)-R1	AAT CAC GAG GAG ATA CTC CAG TTA CCA ATG CTT AAT CAG TGA		
Down(InvF-bla)-F1	TCA CTG ATT AAG CAT TGG TAA CTG GAG TAT CTC CTC GTG ATT		
NotI (InvF)-R1	ATA TGC GGC CGC CGA TGA CAT ACC ATT CAT		
InvF-F	ATG CTG AAT CCT GTT GAA		
InvF-R	AAT CAC GAG GAG ATA CTC		
114upXhoI(invF)-F1	TGC AAT GAA TGC CAT CTG CT		

Supplementary Table 2. Primers used for qRT-PCR analysis

Name	Sequence (5'-3')	Target genes	References
cspaL-qRT-F	GGG CAT ATT TAC CTG AGC CAT A	*spaL* (chromosome)	This study
cspaL-qRT-R	CTG TCG ATG TTC TGG TGT AGT C		
pspaL-qRT-F1	GAT CTG GTT CAT CTT CAC CTT CT	*spaL* (plasmid)	
pspaL-qRT-R1	GAC AGT TTG CCT CGG ATA CTT		
qRT-invE-F	GAT GCT CGC CAA GCT GAG GC	*invE*	
qRT-invE-R	TGC GCC ATC GCT GCA GAC AT		
qRT-invA-F	ACG TGT GGC TGA GGT TGC AGC	*invA*	
qRT-invA-R	GCA TCA GCA TCA ATG ATC CCT G		
qRT-spaK-F	TGA TGA GGA TGT GTG GTT ATG G	*spaK*	
qRT-spaK-R	CAT CCC GCC ATT AAT GCG CGC A		
sicA-qRT-F	GAT CTT TAT GCC GTG GCT TTC	*sicA*	
sicA-qRT-R	GCT TTC GCA GCT TTA CTC ATC		
qRT-sipB-F	TGC TAG CAT AGT TGG TGC CGT	*sipB*	
qRT-sipB-R	TGA GGT GCC AGT AGC AGC CT		
sipC-qRT-F	CGG TAG AAG CGT CAT GAG TAA C	*sipC*	
sipC-qRT-R	GTT AGG CGC GAG CAA TAA GA		
qRT-sipD-F	GGA CAT CAT GGT GTG TAC TTG T	*sipD*	
qRT-sipD-R	TCT CTA ATG TTG GTC GAT CAC C		
spaS-qRT-F	AAG TAT TCA GCG AGT GCA TCT AA	*spaS*	
spaS-qRT-R	TGT GGC AAT TGT TTG GGA AAG		
prgI-qRT-F	AGA TGA TGT TTC TCG TAC CTT TGA	*prgI*	
prgI-qRT-R	TGT GCT AAT AAT GCA GGA TCT GA		
recA-qRT-F	GCA GAA CAT GCC CTT GAT CC	*recA*	
recA-qRT-R	TGT TCA CCC GTA TCT GGC TG		

Supplementary Table 3. Transferability of pJH-1 from strain JH-1 to other p*spaL* or *invF* gene-negative Providencia and other related enteric bacteria

Bacteria (n)	No. of strains acquired pJH-1	Transformation efficiency (/recipient)
P. rustigianii (1)	1	$\leq 10^{-5}$
P. alcalifaciens (2)	0	$\geq 10^{-7}$
P. heimbachae (1)	0	$\geq 10^{-7}$
P. rettgeri (1)	1	$\leq 10^{-5}$
P. stuartii (1)	0	$\geq 10^{-7}$
E. coli (4)	1	$\leq 10^{-3}$
Salmonella enterica (2)	0	$\geq 10^{-7}$
Shigella flexnerii (2)	0	$\geq 10^{-7}$
Pseudomonas aeruginosa (2)	0	$\geq 10^{-7}$

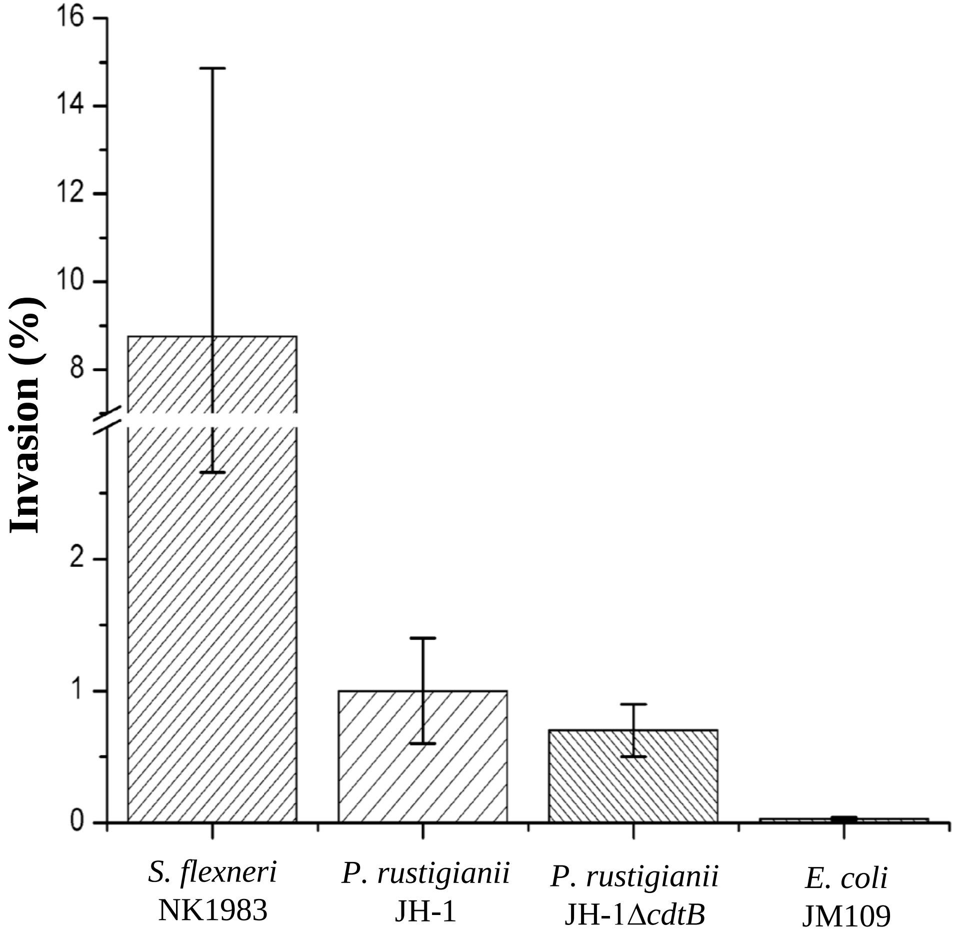

Supplementary Fig. 1. Invasion of HeLa cells by P. *rustigianii* strain JH-1 (WT and Δ*cdtB*). Invasion assay was performed under gentamicin protection at MOI = 1.0. The data showed in the figure includes Mean ± SD of an experiment done in triplicates. *S. flexneri* strain NK1983 and *E. coli* strain JM109 were used as the positive and negative controls, respectively. Experiment was repeated at least 3 times.

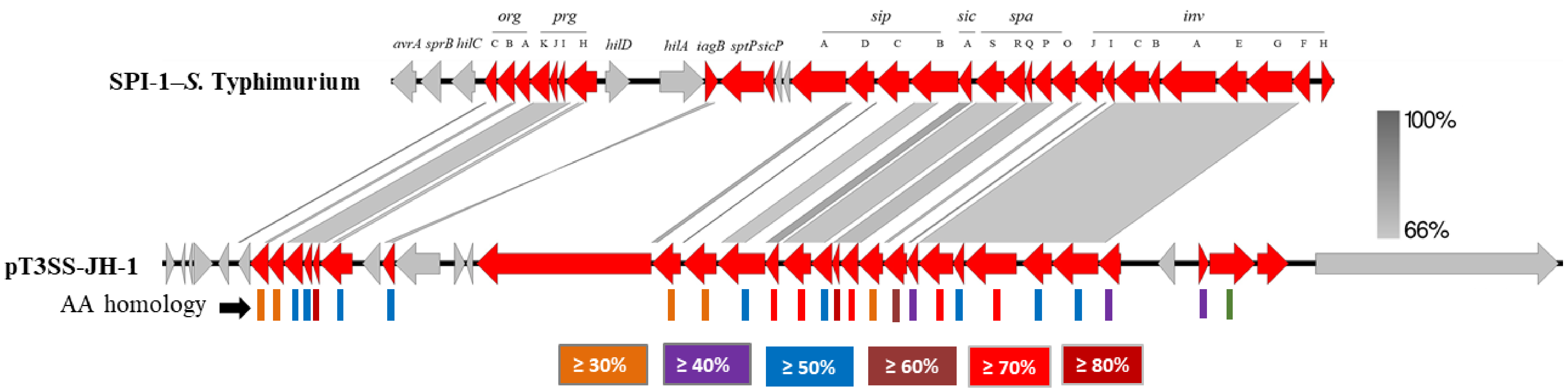

Supplementary Fig. 2. Linear comparison of pT3SS of strain JH-1 with T3SS-1 on SPI-1 of *S.* Typhimurium strain LT2 (Accession No. AE006468.2). T3SS related genes are indicated by red arrows. Colored bars underneath indicates deduced amino acid (AA) sequence homology between the respective sequences (≥ 30% to ≥ 80%). homology under the locus_tag. The homology was calculated based on blastx homology against *S. enterica* ser. typhimurium (taxid:90371). The Figure was generated on Easyfig 2.2.5 (http://mjsull.github.io/Easyfig/).

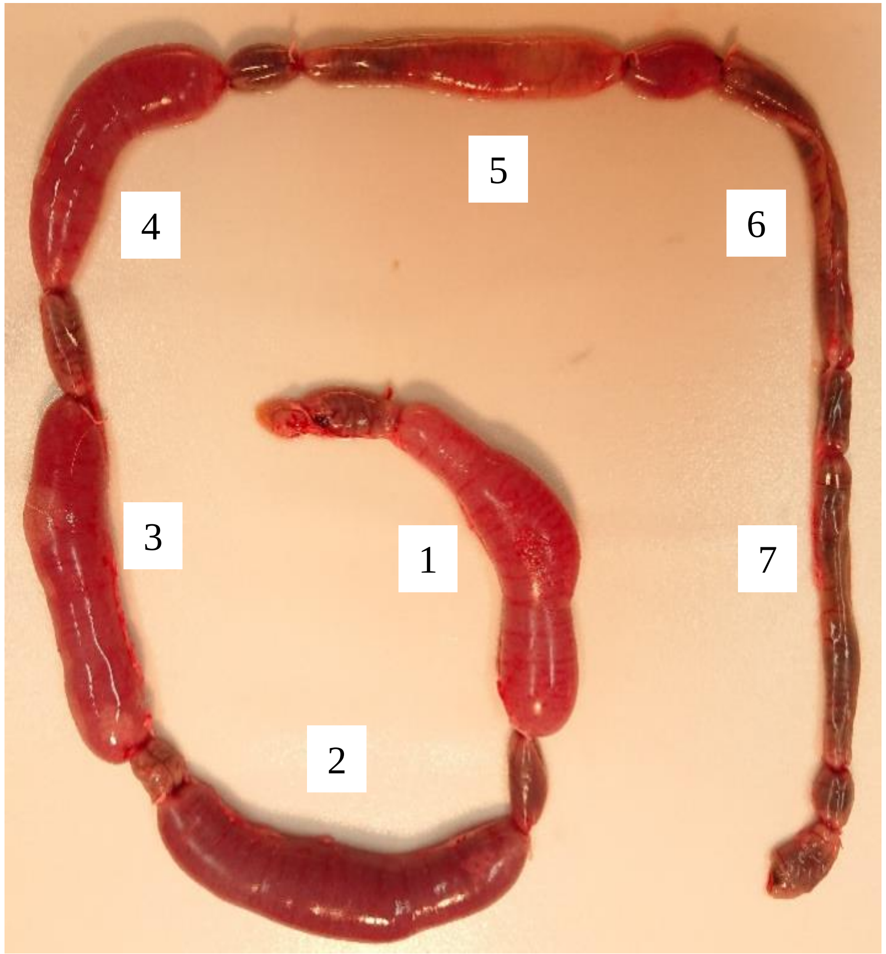

Supplementary Fig. 3. Representative picture showing fluid accumulation in the rabbit ileal loops induced after 16 h of challenge with strain JH-1 [wild type (WT) and *spaL* mutants]. 1 and 2, WT strain at about 2×10^7 and 2×10^6 cfu, respectively; 3 and 4, Δc*spaL* at about 2×10^7 and 2×10^6 cfu, respectively; 5 and 6, Δp*spaL* at about 2×10^7 and 2×10^6 cfu, respectively; 7, PBS (negative control).

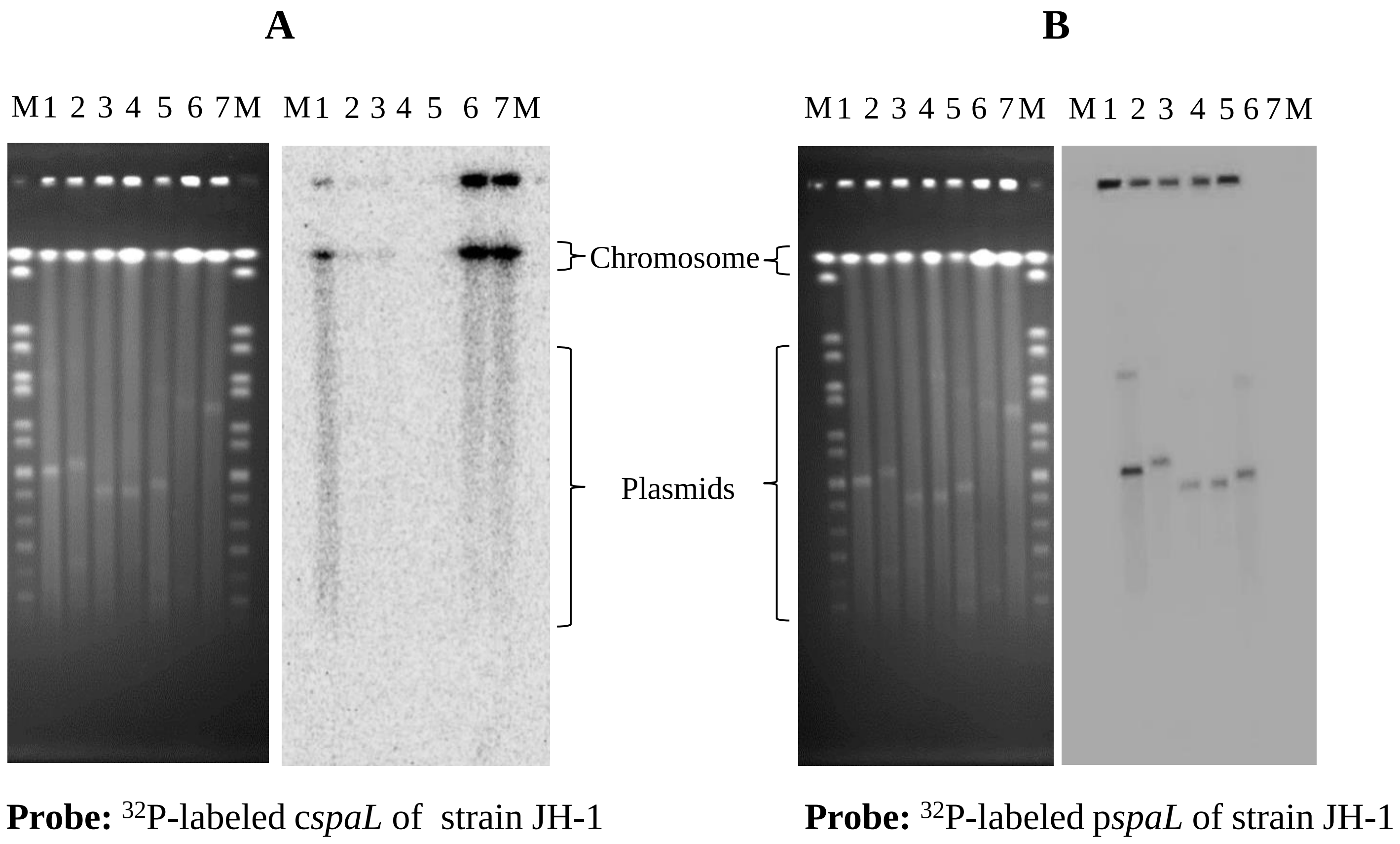

Supplementary Fig. 4. Presence of strain JH-1 *spaL* gene like sequences in other *Providencia* spp. Plasmid profile was analyzed by S1 nuclease-PFGE (left panels of A and B). Subsequently, Southern hybridization was performed to detect *spaL* gene of cT3SS (c*spaL*) (right panel of A) by c*spaL* gene probe and pT3SS (right panel of B) by pT3SS-*spaL* (p*spaL*) gene probe in various strains of *P. rustigianii* and *P. alcalifaciens*. Lanes: 1, *P. rustigianii* strain JH-1; 2, *P. alcalifaciens* strains AH-31; 3, AS-1; 4, P6400; 5, F-90-2004; 6, *P. rustigianii* strains GTC1504; 7, Ch2012-M11; M, molecular size markers prepared by *Xba*I digestion of *S.* Braenderup strain H9812.

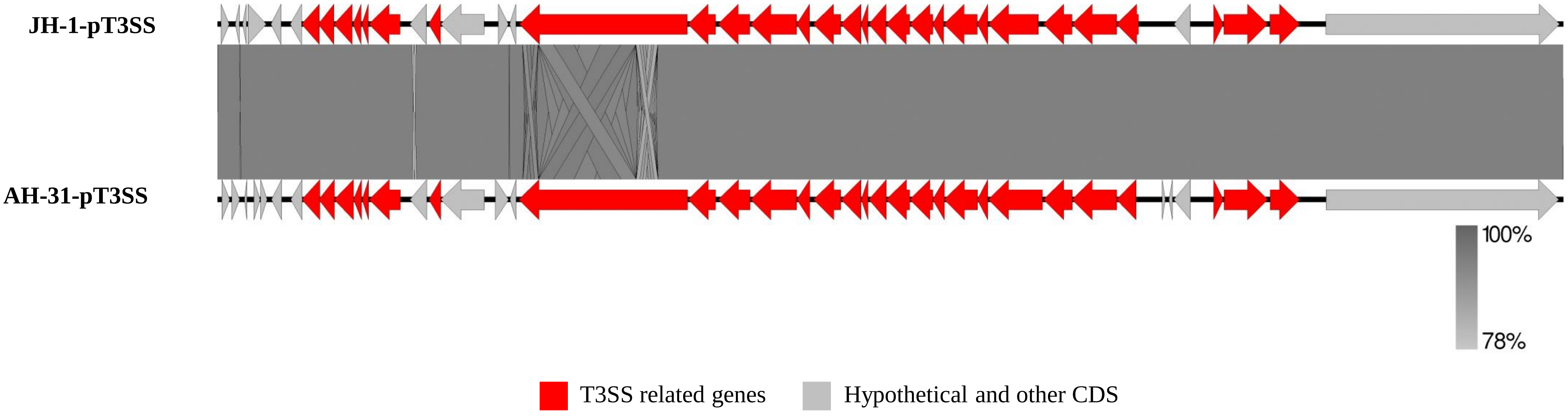

Supplementary Fig. 5. Linear comparison of T3SS sequences in *P. rustigianii* strain JH-1 (pJH-1) and *P. alcalifaciens* strain AH-31 (pAH-31). pAH-31 sequences were derived through reference mapping of raw reads of strain AH-31 against pJH-1. Different color arrows indicates types of genes as denoted in the figure legends.

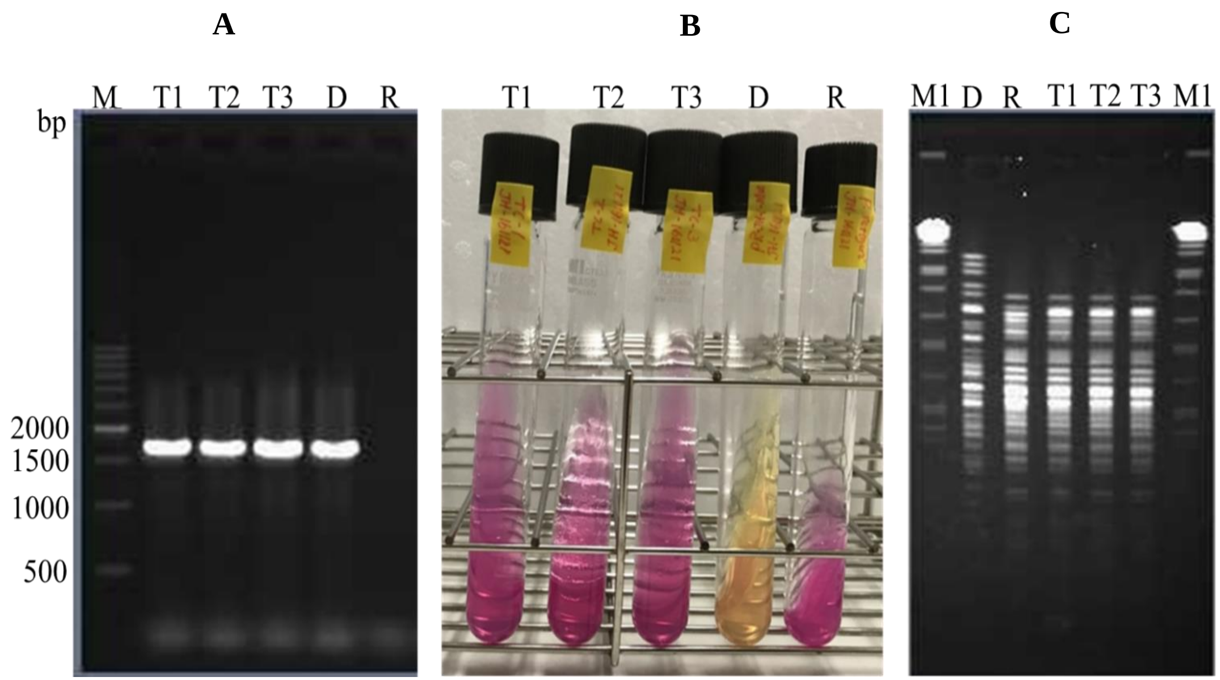

Supplementary Fig. 6. Horizontal transfer of plasmid (pJH-1) from *P*. *rustigianii* strain JH-1 to *P*. *rettgeri* strain GTC1263 through conjugation. Transfer of pJH-1 to recipient strain was confirmed by PCR for the mutated p*spaL* gene (A), urease test (B) and PFGE pattern analysis by *Sma*I digestion (C). Lanes: D, *P*. *rustigianii* strain JH-1 Δp*spaL* (donor); R, *P*. *rettgeri* strain GTC1263 (recipient); T1 to T3, transconjugants; M, 500 bp DNA ladder (TaKaRa); M1, molecular size markers prepared by *Xba*I digestion of *S*. Braenderup strain H9812.

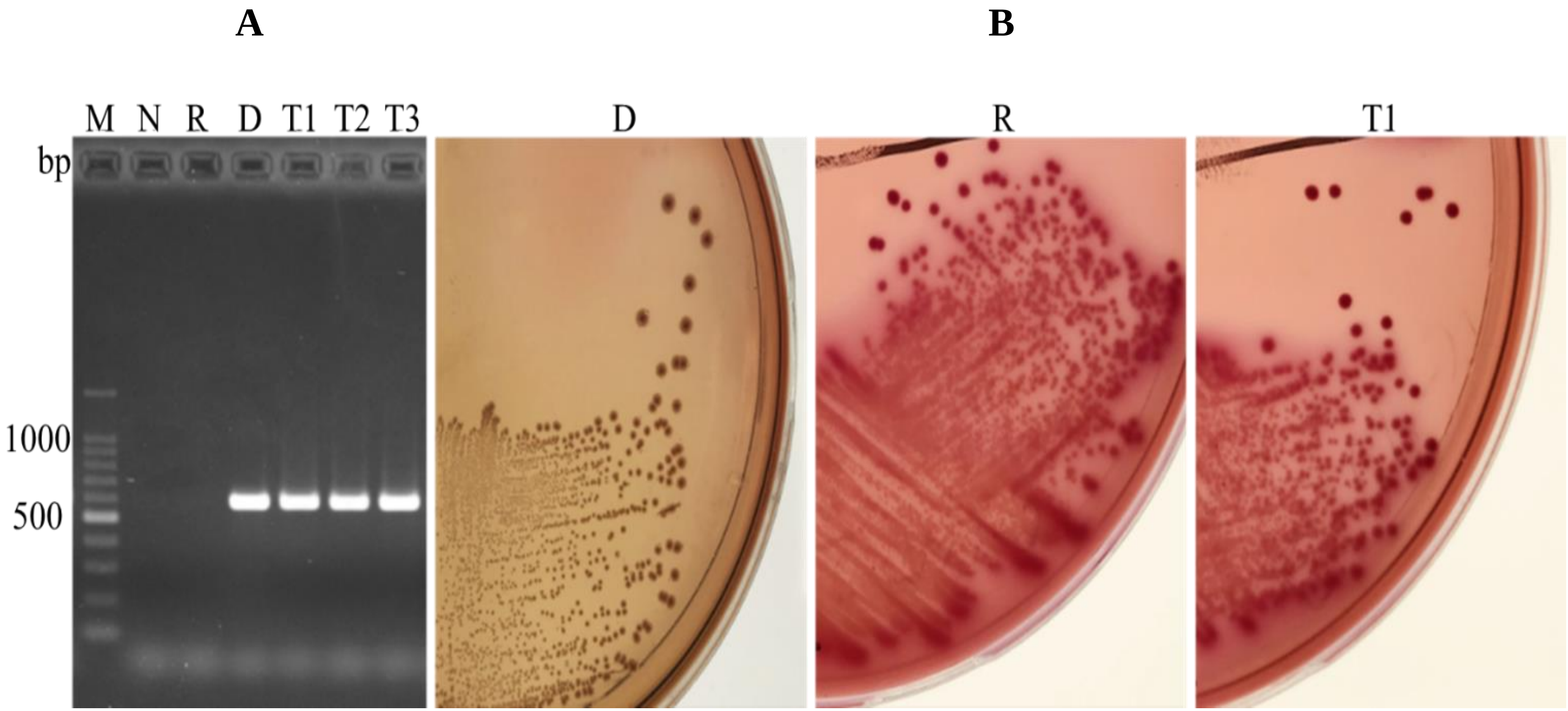

Supplementary Fig. 7. Horizontal transfer of plasmid (pJH-1) from *P. rustigianii* strain JH-1 to *E. coli* KC95 through conjugation. Transfer of pJH-1 to recipient strain was confirmed by PCR for *cdtB* (A) and colony colors on MacConkey agar plates (B). Lanes: D, *P. rustigianii* strain JH-1 Δ*invF* (donor); R, *E. coli* KC95 (recipient); T1 to T3, transconjugants; M, 100 bp DNA ladder (TaKaRa).
